# A Diagnostic Algorithm for Reconstructing the Direction of Gunshots Using OsiriX and Maya in Living Patients: A Forensic Radiology Approach

**DOI:** 10.3390/diagnostics16020344

**Published:** 2026-01-21

**Authors:** Ginevra Malta, Stefania Zerbo, Tommaso D’Anna, Simona Pellerito, Antonina Argo, Mauro Midiri, Giuseppe Lo Re, Francesca Licitra, Angelo Montana

**Affiliations:** 1 Department of Health Promotion, Mother and Childcare, Internal Medicine and Medical Specialties (PROMISE), University of Palermo, 90129 Palermo, Italy; stefania.zerbo@unipa.it (S.Z.); tommaso.danna@students.uniroma2.eu (T.D.); simona.pellerito00@gmail.com (S.P.); antonella.argo@unipa.it (A.A.); mauro.midiri@unipa.it (M.M.); 2 Department of Biomedicine and Prevention, University of Rome “Tor Vergata”, 00133 Rome, Italy; 3 Radiology Unit, Department of Biomedicine, Neuroscience and Advanced Diagnostics, University of Palermo, 90127 Palermo, Italy; giuseppe.lore01@unipa.it; 4 Department of Biomedical Sciences and Public Health, Marche Polytechnic University, 60126 Ancona, Italy; flicitra10@gmail.com

**Keywords:** forensic radiology, gunshot wounds, trajectory reconstruction, CT, OsiriX, Maya, 3D modeling

## Abstract

**Background/Objectives:** Gunshot wounds in living patients present significant challenges from both a clinical and a forensic perspective. Understanding the exact trajectory of a bullet is crucial not only for guiding treatment but also for providing reliable documentation in legal settings. This work introduces a practical diagnostic workflow that combines OsiriX (V. 14.1.1), a DICOM viewer with advanced 3D tools, with Autodesk Maya, a modeling platform used to recreate the external shooting scene. **Methods:** CT scans obtained with multidetector systems were analyzed in OsiriX using a structured, seven-step process that included multiplanar reconstructions, 3D renderings, and region-of-interest tracking. The reconstructed trajectories were then exported to Maya, where they were integrated into a virtual model of the shooting scene to correlate internal findings with the incident’s external dynamics. **Results:** The workflow allowed precise identification of entry and exit points, reliable reconstruction of bullet paths, and effective 3D visualization. While OsiriX provided detailed information for clinical and radiological purposes, the use of Maya enabled simulation of the external scene, improving forensic interpretation and courtroom presentation. The procedure proved reproducible across cases and compatible with emergency timelines. **Conclusions:** The combined use of OsiriX and Maya offers a reproducible and informative method for analyzing gunshot wounds in living patients. This approach not only supports surgical and diagnostic decisions but also enhances the forensic value of radiological data by linking internal trajectories to external shooting dynamics. Its integration into trauma imaging protocols and forensic workflows could represent a significant step toward standardized ballistic documentation.

## 1. Introduction

Gunshot wounds (GSWs) represent a significant proportion of trauma cases worldwide and are frequently associated with high morbidity and mortality [1]. The pathophysiology of ballistic injuries is complex, shaped by multiple variables including projectile velocity, angle of penetration, tissue density, and the involvement of bone structures [2]. From a clinical perspective, GSWs pose major diagnostic challenges, as accurate assessment of the bullet’s internal trajectory and its potential interaction with vital organs is essential not only for surgical planning and therapeutic management but also for the precise documentation of injury patterns with medicolegal relevance [3].

In forensic medicine, bullet trajectory reconstruction plays a pivotal role in understanding the dynamics of shooting incidents. Determinations such as the directionality of the shot (e.g., anteroposterior, craniocaudal), the relative positioning of the shooter and the victim, and the number of projectiles involved often rely on radiological evidence. In recent years, post-mortem computed tomography (PMCT) has become the reference standard for non-invasive analysis of bullet paths during forensic autopsy [4]. However, the growing demand for accurate evaluation in living patients—driven by advances in trauma care and increasing legal scrutiny—has highlighted the need for reproducible in vivo imaging methodologies [5].

Computed tomography (CT) remains the cornerstone imaging modality for penetrating trauma. The advent of high-resolution multidetector CT (MDCT) systems has enabled the acquisition of thin-slice datasets that can be processed into highly detailed reconstructions of internal injuries. The diagnostic potential of CT is further enhanced when combined with advanced post-processing platforms that support three-dimensional visualization, trajectory estimation, and modeling of projectile paths [6].

Among these, OsiriX, a macOS-based DICOM viewer and image analysis platform, offers a wide range of functionalities particularly suited for ballistic applications. These include multiplanar reconstruction (MPR), volume rendering (VR), region-of-interest (ROI) annotation, and even Fly-Thru navigation [7]. Together, these tools allow detailed tracking of bullet tracts across anatomical planes, creation of trajectory vectors, and calculation of entry-to-exit angles relative to anatomical landmarks, while also correlating internal imaging findings with external clinical examination.

Beyond radiological reconstruction, an additional dimension can be achieved by integrating imaging data into three-dimensional modeling environments, such as Autodesk Maya [8]. This approach enables the transposition of CT-derived trajectories into a simulated shooting scene, thereby correlating internal bullet paths with external spatial dynamics. Such integration provides a comprehensive representation of both clinical injuries and forensic context, offering enhanced clarity for trauma management and courtroom presentation.

However, such simulated scenes, based exclusively on CT-derived internal trajectories, should be seen as tools for visualization and hypothesis testing. They are not deterministic reconstructions of real-world shooting dynamics. External scene modeling always depends on independent forensic inputs, such as crime scene investigation data, witness statements, ballistic evidence, and body positioning at the time of discovery.

The aim of this study is to propose a structured, reproducible diagnostic algorithm. This algorithm first reconstructs projectile trajectories using OsiriX and then models the three-dimensional scene in Maya. The primary objective of this workflow is to enable forensic–radiological reconstruction in living patients, providing robust and reproducible medico-legal documentation based on retrospectively acquired imaging data.

By transforming CT datasets into persistent three-dimensional models, the workflow overcomes some intrinsic limitations of image-based analysis in living subjects. This process generates trial-relevant documentation that can be reviewed and reassessed over time.

A secondary benefit of the proposed approach is its potential clinical utility. It may aid in surgical planning and diagnostic support by enhancing visualization of projectile paths and their possible anatomical implications.

## 2. Materials and Methods

### 2.1. Study Design and Scope

This study outlines a diagnostic workflow for reconstructing gunshot wound (GSW) trajectories in vivo using OsiriX (V. 14.1.1), a macOS DICOM viewer, and subsequently integrating them into Autodesk Maya for external scene modeling. The workflow was refined through retrospective analysis and applied to clinical cases of firearm injury. Validation occurred in cases with complete radiological datasets, using surgical correlation and, when available, forensic documentation.

### 2.2. Imaging Acquisition Protocol

All CT examinations included in this workflow were acquired using multidetector computed tomography (MDCT) scanners capable of thin-slice reconstruction. To ensure optimal spatial resolution and consistency for 3D post-processing, the following acquisition parameters were standardized across the reviewed cases:

• Scanner type: minimum 128-slice MDCT systems;
• Slice thickness: 0.5–1.0 mm;
• Reconstruction interval: 0.5 mm;
• Scan range: tailored to the anatomical region of suspected injury (thorax, abdomen, head, or extremities);
• Contrast protocol: biphasic contrast-enhanced imaging performed when vascular or solid organ injury was suspected (arterial and venous phases);
• Patient positioning: supine, with arms either alongside the body or raised above the head depending on clinical status; orientation markers verified during post-processing.

### 2.3. Inclusion and Exclusion Criteria

The workflow was applied exclusively to cases that satisfied a strict set of inclusion criteria. Eligible patients were those with confirmed gunshot wounds who had undergone multidetector CT within six hours of the traumatic event. Each case had to demonstrate a clearly visible entry wound, a definable projectile path, and either a retained bullet or an unequivocally identifiable exit wound. To avoid confounding variables, patients who had already undergone surgical intervention or projectile removal prior to imaging were excluded. In addition, only scans free from significant motion artifacts, which might compromise image quality and interpretation, were considered suitable for analysis.

Cases were excluded from the workflow if they did not meet the necessary technical or clinical requirements for accurate reconstruction. Patients who arrived more than six hours after sustaining the gunshot injury were excluded, as delayed imaging could compromise the visualization of soft tissue tracts due to evolving hemorrhage, edema, or early surgical manipulation. Likewise, individuals who had already undergone operative procedures, such as debridement or projectile removal prior to CT acquisition, were not considered suitable, since these interventions could alter the native trajectory or anatomical landmarks. Scans affected by severe motion artifacts or pronounced metallic streak artifacts were also excluded, as they significantly reduce the ability to reconstruct reliable trajectories. Finally, patients with incomplete imaging datasets, inadequate coverage of the anatomical region of interest, or insufficient clinical documentation to verify entry and exit sites were omitted from the analysis.

### 2.4. Software and Tools

All imaging datasets were analyzed using OsiriX MD (v.12 or later) or, where available, OsiriX Lite with reduced functionality. The following tools were employed within OsiriX:

• Multiplanar Reconstruction (MPR): for dynamic realignment of imaging planes;
• 3D Volume Rendering (VR): for global anatomical overview and visualization of metallic fragments;
• Region of Interest (ROI) tools: to annotate entry and exit points and trace trajectory lines;
• Fly-Thru navigation: to simulate an endoscopic view of the projectile tract;
• Angle and distance measurements: to calculate trajectory vectors in anatomical space.

Following trajectory reconstruction in OsiriX, the resulting 3D vectors and annotated datasets were exported in DICOM 3D or STL format. The use of these formats preserved the original spatial resolution and metric scale derived from CT metadata, allowing the trajectory vectors to be imported into Autodesk Maya with preservation of the original metric scale.

The trajectory vectors were defined within the patient-based anatomical reference frame derived from the CT acquisition and were visualized in Maya without modification.

Within Maya, these data were integrated into a virtual reconstruction of the shooting scene, allowing for correlation between internal trajectories and external spatial dynamics.

### 2.5. Diagnostic Algorithm for Trajectory Reconstruction

A standardized seven-step algorithm was developed and systematically applied to each case:

**Step 1: Image Preprocessing and Orientation Correction**. The workflow begins with the importation of DICOM datasets into the OsiriX environment. Patient orientation is then carefully adjusted to correspond with true anatomical planes in the axial, sagittal, and coronal views. At this stage, overall image quality is assessed, and an initial survey is performed to detect fractures, intraparenchymal gas, or metallic fragments that may indicate the projectile path.

**Step 2: Localization of Entry and Exit Wounds**. The next step involves the identification of radiological signs corresponding to external breaches, such as subcutaneous emphysema, hematoma, or soft tissue disruption. Bullet entry and exit points are confirmed by tracing linear tissue channels, and each site is annotated with region-of-interest (ROI) markers, with their spatial coordinates recorded for further analysis.

**Step 3: Multiplanar Reconstruction (MPR)**. Multiplanar reconstructions are then realigned along the hypothesized trajectory, enabling the radiologist to follow the bullet tract throughout the dataset. This process facilitates the recognition of secondary lesions, including bone fractures, cavitation zones, and vascular involvement. Using the measurement tools within OsiriX, the orientation of the projectile is calculated in relation to anatomical planes, whether craniocaudal, anteroposterior, or oblique.

**Step 4: 3D Volume Rendering (VR)**. Three-dimensional volume rendering is subsequently applied using presets optimized for bone or metallic density. By adjusting opacity and brightness, soft tissue interference is minimized, allowing clear visualization of projectile fragments, bone splintering, and potential ricochet points. Volume rotation further assists in correlating internal trajectories with external entry and exit wounds.

**Step 5: Fly-Thru Navigation (Virtual Tract Endoscopy)**. To complement these reconstructions, the Fly-Thru navigation tool is employed, providing an endoscopic-like perspective of the suspected bullet path. This virtual navigation, adjusted for transparency and viewing angles, allows the projectile’s course through tissues to be simulated and documented through captured images or video sequences. These outputs serve both clinical decision-making and forensic presentation.

**Step 6: Bullet Path Modeling and Measurement**. Trajectory modeling is then carried out using ROI lines or spline tools to generate a three-dimensional representation of the projectile’s path. The starting point (entry wound) and endpoint (exit site or retained fragment) are marked, and trajectory angles are measured across all anatomical planes. Any deviations, including deflection, ricochet, or fragmentation, are documented and analyzed.

**Step 7: Documentation, Reporting, and Scene Reconstruction**. The final step consists of structured documentation and comprehensive reporting. This includes a description of entry and exit wound locations, the trajectory path and its orientation, involvement of organs, bones, or vasculature, as well as any evidence of deflection or secondary fragmentation. Annotated 2D images and 3D renderings are generated, and the reconstructed vectors are exported for medico-legal archiving. Importantly, the dataset is also integrated into Autodesk Maya, where the internal trajectories are combined with external body positioning to simulate the shooting scene. This integration provides a more complete forensic visualization, linking internal ballistic paths to external dynamics and significantly enhancing courtroom presentation.

**Figure 1:**
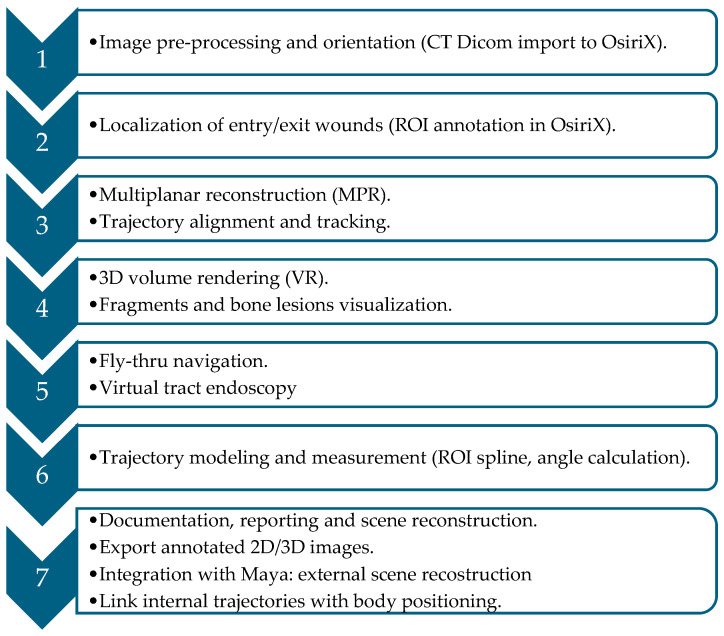
Stepwise workflow for bullet trajectory reconstruction using OsiriX and Maya, from CT-based internal modeling to external scene simulation for clinical and forensic applications.

These steps are summarized in Figure 1.

### 2.6. Ethical Considerations

All imaging data analyzed in this study were obtained from routine clinical practice and subsequently anonymized prior to review. The reconstruction workflow was applied retrospectively and did not influence patient management or therapeutic decision-making. As such, the study relied exclusively on pre-existing datasets and did not involve any procedures beyond those required for standard diagnostic care. The analysis was performed under the supervision of board-certified radiologists and forensic experts, ensuring adherence to accepted professional standards.

## 3. Results

### 3.1. Case Series Overview

Between January and October 2024, the diagnostic algorithm was retrospectively tested on a cohort of ten patients (eight males and two females; mean age 32.8 years, range 19–54) who presented with firearm-related injuries at a Level I Trauma Center. All individuals underwent multidetector CT (MDCT) within six hours of admission, and in every case, either a retained projectile or clearly recognizable entry and exit wounds were identified. The imaging datasets were independently assessed by two radiologists, one with more than a decade of experience in forensic imaging, using the OsiriX MD platform. Cases are summarized in Table 1.

### 3.2. Algorithm Performance

The application of the algorithm yielded successful trajectory reconstructions in all ten cases examined, supporting the feasibility of the proposed workflow. High inter-observer agreement was observed, with complete agreement between the two radiologists in 90% of cases, while minor discrepancies in angle estimation—never exceeding 5 degrees—were observed in the remaining cases.

The mean time required to perform a full reconstruction was 18.4 min, with values ranging from 11 to 27 min depending on the anatomical region and complexity of the injury.

In terms of forensic relevance, the reconstructed trajectories were qualitatively assessed as having high utility in seven cases, particularly where well-defined paths allowed for effective visualization in judicial settings. In two cases, the utility was considered moderate, largely due to partial deflection of the projectile or ambiguities in soft tissue involvement. Only one case was categorized as having low forensic value, corresponding to a straightforward through-and-through extremity injury with limited interpretive complexity.

### 3.3. Imaging and Documentation Outputs

In all cases, annotated three-dimensional volume renderings and multiplanar reconstruction (MPR) images were successfully generated and exported, allowing for comprehensive documentation of the reconstructed trajectories. The bullet paths were consistently visualized in relation to the principal anatomical planes, with trajectory vectors superimposed to facilitate spatial interpretation. In three instances, the Fly-Thru tool produced particularly effective virtual endoscopic views of the projectile tract, which were subsequently incorporated into courtroom presentations to enhance evidentiary clarity. Furthermore, the reconstructed vectors were exported into Autodesk Maya, where they were integrated into external body positioning and scene modeling. This additional step provided a more complete forensic representation, linking internal ballistic trajectories with the external shooting dynamics. Taken together, these findings confirm the feasibility and reproducibility of the proposed workflow, underscoring its clinical and forensic value, particularly in complex thoracoabdominal and cranial injuries.

**Figure 2:**
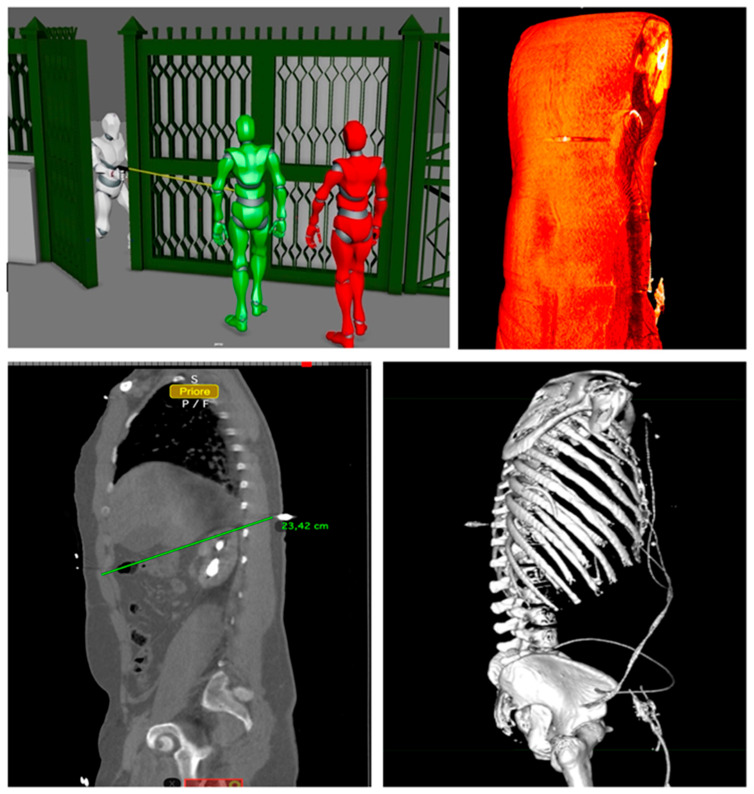
Combined radiological and scene-based reconstruction of a gunshot trajectory. Multidetector CT images processed in OsiriX (**bottom left**: sagittal MPR with trajectory measurement; **bottom right**: 3D volume rendering of the thorax; **top right**: high-density visualization of the projectile tract) are integrated with Autodesk Maya scene modeling (**top left**), illustrating the external positioning of the victim (green) and one of the shooters (red) at the moment of discharge. This multimodal workflow enables correlation of internal ballistic paths with external spatial dynamics, enhancing both clinical interpretation and forensic visualization.

**Figure 3:**
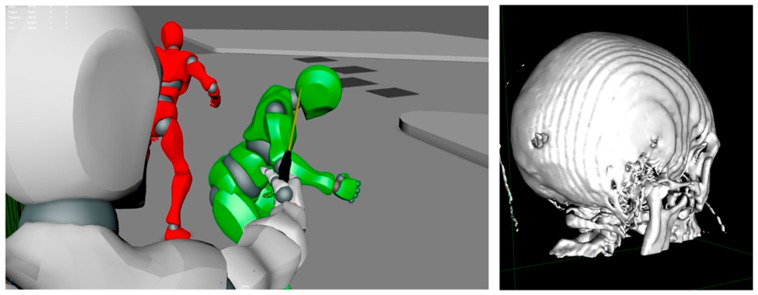
Example of combined reconstruction in a cranial gunshot wound. On the left, Autodesk Maya scene modeling illustrates the external dynamics of the shooting, depicting the shooter (red) and the victim (green) now of impact. On the right, a 3D CT reconstruction obtained in OsiriX shows the skull with extensive fracture lines and bone fragmentation consistent with the projectile’s entry. The integration of radiological data and scene simulation provides a comprehensive framework for clinical assessment and forensic interpretation.

Most significant images are reported in Figure 2 and Figure 3.

## 4. Discussion

This study demonstrates that bullet trajectory reconstruction in living patients using OsiriX is both feasible and reproducible, providing valuable information for clinical decision-making and forensic interpretation. The proposed seven-step workflow was successfully applied across a heterogeneous series of gunshot injuries, encompassing different anatomical regions, projectile behaviors, and levels of clinical complexity. The results support the diagnostic utility of this approach and underline its potential for systematic integration into trauma imaging protocols and forensic investigations.

In all 10 cases analyzed, the algorithm enabled accurate reconstruction of bullet trajectories, directly supporting clinical interpretation and surgical planning. Complex injuries involving the thoracoabdominal junction, the pelvis, and the cranial vault were successfully modeled, facilitating the early identification of potential multi-organ involvement. In one representative case, a retained projectile in the pelvis with an oblique caudo-medial course suggested a possible transperitoneal path, which proved crucial in guiding operative exploration and management.

The mean reconstruction time of 18.4 min confirmed the workflow’s compatibility with the demands of emergency settings. The ability to assess trajectories across multiple planes and within three-dimensional renderings provided clinicians with a clearer overview of injury patterns, particularly in thoracic and abdominal GSWs, where critical damage may remain inconspicuous on axial slices alone [9].

Beyond its clinical applicability, the proposed workflow demonstrated substantial value in forensic documentation.

While the clinical usefulness of three-dimensional trajectory reconstruction—particularly for surgical planning and diagnostic support—can be readily appreciated, the primary aim of the workflow remains the generation of comprehensive medico-legal documentation in living patients through forensic-radiological reconstruction.

This distinction is reflected in the study results: in 7 out of 10 cases, the reconstructed trajectories were considered highly relevant for medico-legal purposes, especially when the projectile path traversed multiple anatomical planes or involved ricochets and bone deflection [10].

Importantly, the forensic application of trajectory reconstruction requires different standards of accuracy, validation, and evidentiary robustness compared to purely clinical use. These requirements justify the emphasis placed on reproducibility, traceability, and post hoc reassessment rather than real-time clinical decision-making.

For example, one case revealed a left-to-right trans-thoracic trajectory associated with rib fractures and posterior vertebral involvement, findings that could substantiate external wound mapping and provide key information on victim positioning and shooting angle.

In three cases, the Fly-Thru tool produced immersive, endoscopic-like visualizations of the projectile tract, which proved especially effective in courtroom settings and during expert testimony. Importantly, integrating reconstructed vectors into Autodesk Maya further enhanced this value, enabling the creation of external scene simulations that correlated internal trajectories with body positioning and spatial dynamics. This combination provided forensic experts with a powerful means of illustrating complex injury mechanisms in a manner both anatomically accurate and accessible to non-specialist audiences, including judges and jurors.

Moreover, preserving CT datasets and reconstructed trajectories ensures the availability of a fixed digital record of injury morphology. This feature is particularly relevant for long-term forensic follow-up, as surgical repair and healing processes may later alter the original wound characteristics [11]. By exporting trajectories into Maya, the workflow creates a stable, reproducible representation that enhances evidentiary reliability in delayed legal proceedings.

Within this context, it is important to position the proposed workflow relative to previously published three-dimensional and PMCT-based reconstruction approaches.

Although several protocols have been described in the literature [5,12,13], relevant differences can be identified when comparing these approaches with the present workflow, particularly with respect to clinical versus post-mortem applicability, metric accuracy, scene reconstruction capabilities, courtroom visualization value, reproducibility, and time efficiency.

Colard et al. [12] reported two cases that employed MSCT combined with three-dimensional reconstruction software and crime scene modeling in SketchUp. While their work demonstrated the potential of integrating radiological data into scene reconstruction, they did not propose a standardized, reproducible methodological framework.

The use of general-purpose three-dimensional software in forensic contexts is now widely regarded as legitimate and of significant communicative value, particularly for educational and courtroom purposes [13].

As emphasized by Riva et al. [5], three-dimensional scene reconstructions are inherently affected by metric uncertainty and should not be assigned the same evidentiary weight as CT-based radiological findings, which directly document internal injuries. Accordingly, external scene reconstruction must always be supported by independent forensic inputs and should be interpreted as an integrative and illustrative tool rather than a deterministic reconstruction.

### Technical Strengths

The high level of inter-observer agreement (90% complete concordance) highlights the robustness and reproducibility of the workflow. Such consistency is critical for trauma care teams and forensic expert panels. Standardized interpretation and reproducibility remain essential in these settings.

The algorithm demonstrated reliable performance in reconstructing linear and mildly oblique trajectories. This held true regardless of whether the projectile was retained or the soft tissue tracts were complex. The ROI and measurement tools available in OsiriX proved adequate for documenting trajectory angles and deviations. Volume rendering offered a clear visualization of ricochet points and secondary fragment dispersion. The subsequent integration of these datasets into Maya provided an additional technical strength. This integration allowed the radiological information to be contextualized within a three-dimensional scene reconstruction. The Maya-based reconstruction is a forensic simulation and illustrative integration. It should not be considered a substitute for ballistic scene reconstruction. Rather, it serves as a didactic and courtroom-communication support tool, not as a standalone source for determining external projectile trajectories.

This dual approach strengthens the clinical workflow and forensic framework, connecting radiological imaging with external ballistic dynamics.

## 5. Limitations and Considerations

Although the findings of this study are promising, several limitations must be acknowledged.

Firstly, the small sample size (10 cases) does not allow inferential statistical conclusions and supports only methodological feasibility and preliminary reproducibility. Accordingly, the present work should be regarded as a pilot proof-of-concept study, rather than a formal validation. Larger prospective, multicenter cohorts will be required to achieve true forensic validation.

The accuracy of trajectory reconstruction depends on the quality of the initial CT acquisition. Motion artifacts, suboptimal contrast timing, and metallic streak artifacts, although relatively limited in the present series, may significantly compromise the precision of visualization. In such circumstances, advanced correction techniques, including iterative reconstruction algorithms, could improve image quality and mitigate these limitations.

Another important consideration relates to the ballistic behavior of projectiles. All cases in this series involved trajectories that were predominantly linear or only mildly oblique. More complex phenomena, such as projectile tumbling, yawing, or secondary fragmentation, may exceed the interpretive capacity of the current algorithm and require additional expertise in wound ballistics. While OsiriX provides reliable visualization of linear tracts, a comprehensive understanding of such atypical behaviors may necessitate integration with external scene reconstruction and experimental ballistic data.

Finally, while OsiriX is widely accepted as a diagnostic tool in radiology, its use in forensic practice varies across jurisdictions. The admissibility of three-dimensional reconstructions in judicial proceedings often depends on validation standards, the quality of documentation, and the qualifications of the reporting expert. The incorporation of Autodesk Maya into the workflow, although advantageous for illustrative purposes, introduces further variability, and its acceptance in courtrooms may require standardized protocols and validation studies.

## 6. Future Implications

The results of this investigation clearly justify integrating OsiriX-based trajectory reconstruction into standard trauma imaging protocols, especially for high-velocity firearm injuries or cases with medicolegal significance. Stakeholders are strongly encouraged to implement this algorithm in their diagnostic workflow. Additionally, expanding its use in larger, multicenter cohorts is essential to confirm its generalizability. Further research should actively pursue the development of automated or artificial intelligence-assisted trajectory modeling to enhance efficiency and reproducibility.

Equally important is the integration of these methods into structured medico-legal reporting frameworks.

The export of reconstructed projectile trajectories into platforms such as Autodesk Maya enables the integration of internal radiological findings with external scene simulations, enhancing the forensic interpretability and evidentiary value of imaging data when appropriately integrated with independent forensic inputs.

While this approach may support clinical work, especially in complex trauma, clinical application was not the main goal of this study. Future research should validate this workflow for clinical decisions, which requires different methods and regulations.

Interdisciplinary collaboration among radiologists, forensic pathologists, ballistic experts, and legal professionals will be essential to formalize these practices and ensure that radiological reconstructions are presented in a scientifically rigorous and legally admissible manner.

## 7. Conclusions

This study introduces a structured diagnostic algorithm for the reconstruction of bullet trajectories in living patients, developed through the application of OsiriX and complemented by external scene modeling in Autodesk Maya. When applied to a series of clinical cases, the workflow demonstrated feasibility, efficiency, and substantial value in both clinical and forensic contexts.

The integration of multiplanar reconstruction, three-dimensional volume rendering, and trajectory modeling within OsiriX enabled accurate visualization and measurement of projectile paths in living patients.

This approach provides a structured and reproducible framework for forensic–radiological reconstruction, generating persistent three-dimensional documentation that can support medico-legal analysis and post hoc reassessment.

The results demonstrate the feasibility of integrating advanced radiological reconstruction techniques into medico-legal workflows based on clinical imaging data.

The subsequent export of reconstructed vectors into Maya further enhanced the forensic dimension of the workflow, enabling the correlation of internal trajectories with external spatial dynamics and providing a powerful tool for medico-legal documentation and courtroom presentation.

While certain limitations remain, including susceptibility to imaging artifacts and the challenges posed by complex projectile behaviors, the proposed algorithm establishes a reproducible framework that can be readily incorporated into trauma imaging protocols. Future research should focus on prospective validation in larger, multicenter cohorts and on the development of standardized protocols for forensic use, ensuring both scientific rigor and legal admissibility.

## Figures and Tables

**Table 1:** Characteristics of the cases examined.

**Case**	**Injury Site**	**Entry Point**	**Exit/Fragment Location**	**Trajectory Angle (°)**	**Bone Involvement**	**Trajectory Reconstruction Successful**	**Forensic Utility**
**1**	Thorax	Left anterior chest	Right paravertebral T8	34 (horizontal)	Rib fractures	Yes	High
**2**	Abdomen	Right lower flank	Bullet retained in pelvis	42 (caudo-medial)	Iliac crest	Yes	High
**3**	Head	Frontal bone	Retained in sphenoid	15 (inferior)	Skull, sinus	Yes	Moderate
**4**	Arm	Lateral humerus	Medial forearm	76 (anteromedial)	Humerus fracture	Yes	High
**5**	Neck	Right SCM region	Left parapharyngeal	12 (transverse)	None	Yes	Moderate
**6**	Chest	Posterior scapula	Bullet retained	60 (inferior)	Scapula, rib	Yes	High
**7**	Abdomen	Epigastrium	Left flank	33 (horizontal)	No	Yes	High
**8**	Lower limb	Thigh (anterior)	Posterior thigh	90 (transfixing)	Femur	Yes	Low
**9**	Pelvis	Perineum	Retained	45 (superior oblique)	Pubic ramus	Yes	Moderate
**10**	Thorax	Right upper chest	Bullet retained T3	25 (posterior oblique)	Rib, vertebra	Yes	High

## Data Availability

The data presented in this study are available on request from the corresponding author. The data are not publicly available due to privacy.
